# Supplementary figures and images for: WHIMP links the actin nucleation machinery to Src-family kinase signaling during protrusion and motility
Source: PLoS Genet. 2020 Mar 20;16(3):e1008694. doi: 10.1371/journal.pgen.1008694 (PMC7112243; doi:10.1371/journal.pgen.1008694)

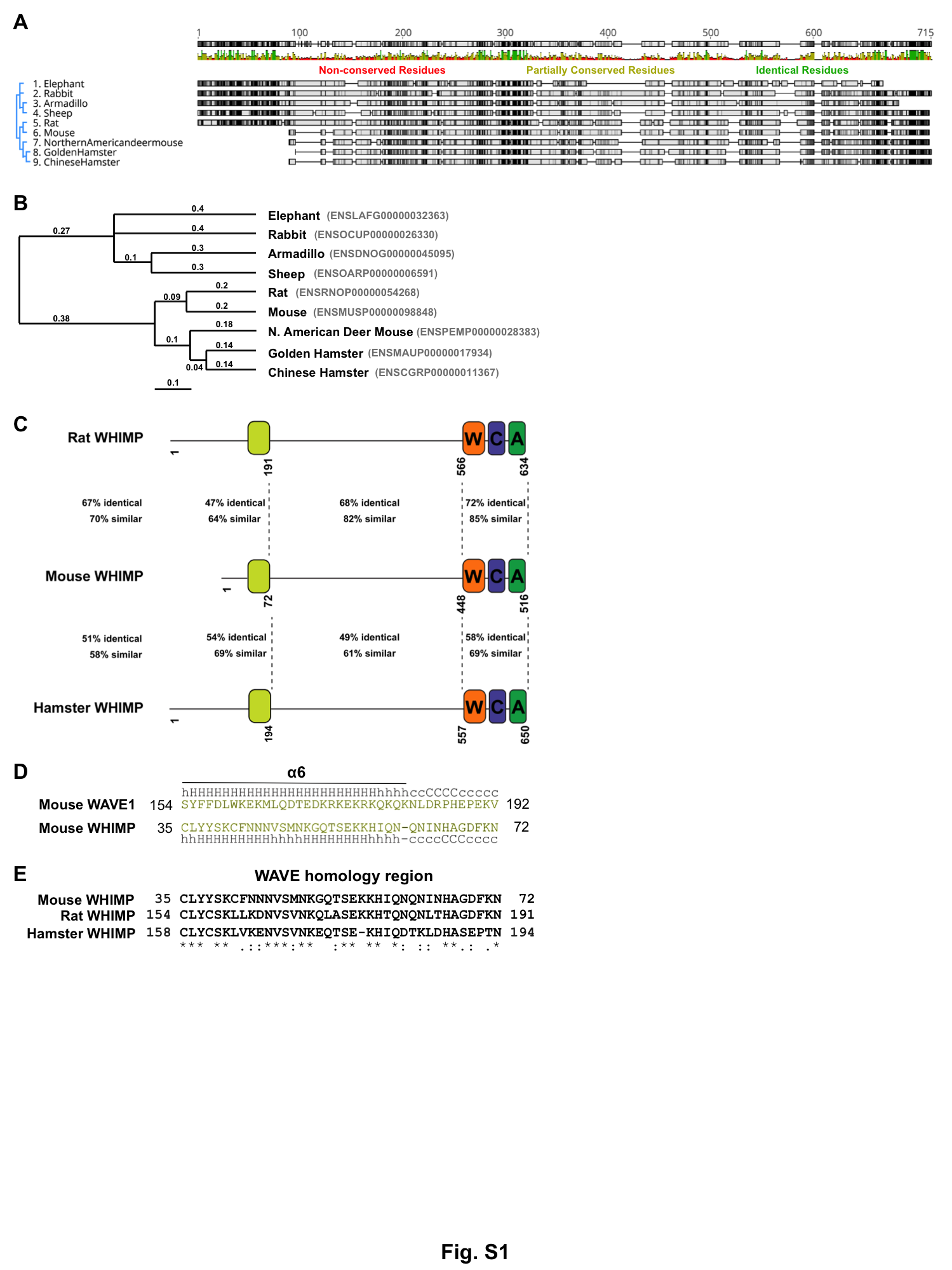

Supplement: S1 Fig — (A) A consensus sequence illustration of WHIMP orthologs aligned using Geneious software is shown. Non-conserved regions are depicted in white, while partially-conserved and identical residues are shown in gray and black. For genomic context, in Mus musculus the Whimp (Gm732) gene is found at X D, while the Was gene is found at X A1.1. (B) A phylogenetic tree of WHIMP was constructed using estimates of maximum likelihood phylogenies (PhyML) from alignments of amino acid sequences generated using Geneious software. UniProt ID numbers are listed next to the common animal names. The scale bar indicates the branch length and amino acid substitutions per site. (C) Primary domain organizations for mouse (M. musculus), rat, and Chinese hamster WHIMP, along with their sequence identities and similarities from EMBOSS Needle are shown. (D) The HHpred algorithm predicts sequence similarity between amino acids 154–192 of the WAVE1 α6 helix and residues 35–72 of WHIMP. H/h = helix and C/c = coil, where uppercase letters represent higher confidence predictions. (E) Multiple sequence alignment of the α6 WAVE homology region of WHIMP from mouse, rat, and hamster is shown. (TIFF) [file pgen.1008694.s001.tiff]

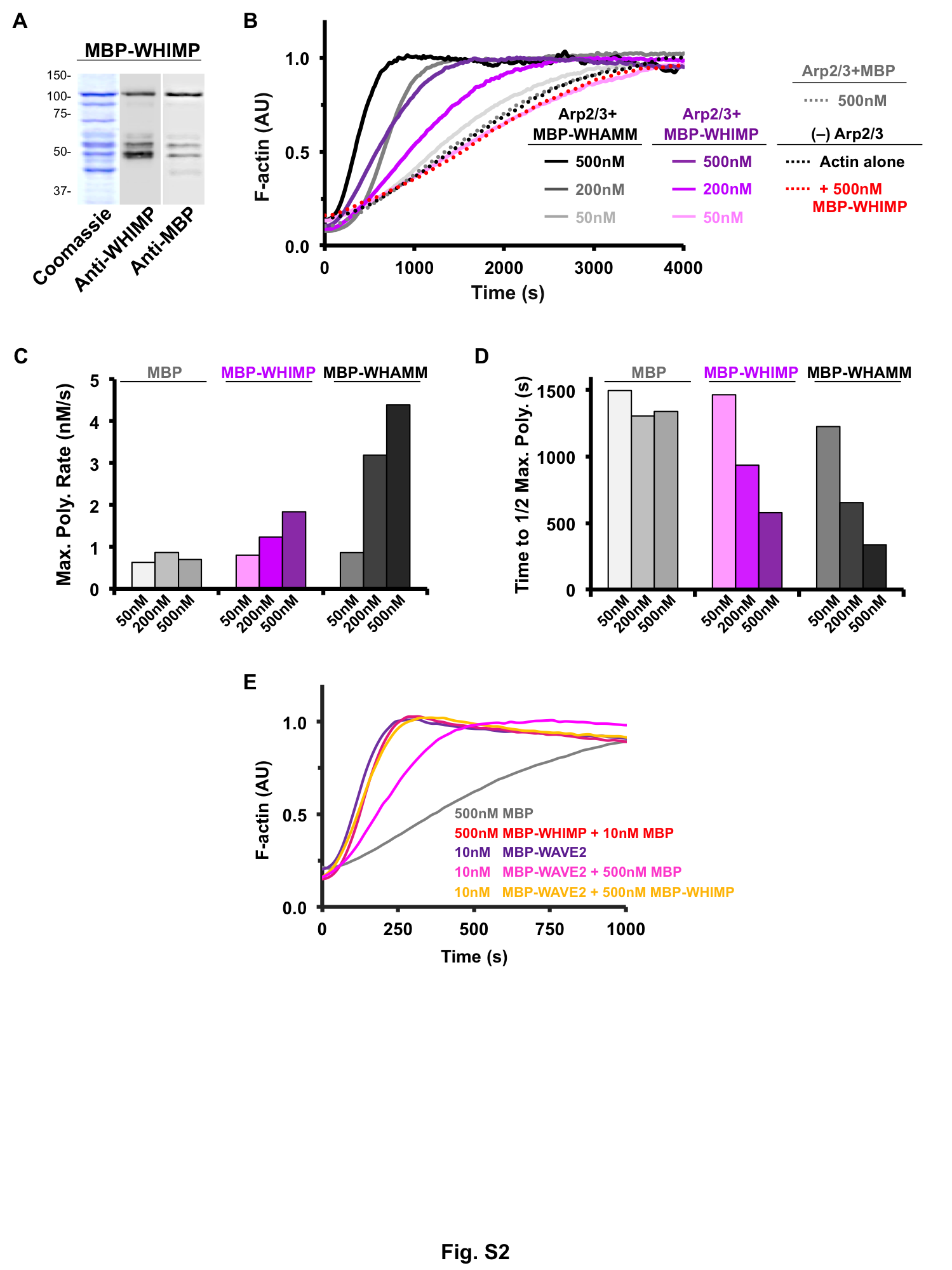

Supplement: S2 Fig — (A) Purified maltose binding protein (MBP) tagged full-length WHIMP was subjected to SDS-PAGE followed by staining with Coomassie blue or immunoblotting with anti-WHIMP or anti-MBP antibodies. (B) Representative actin polymerization assays using 2μM actin, 20nM Arp2/3 complex, and the indicated concentrations of MBP, MBP-WHIMP, or MBP-WHAMM are shown. AU, arbitrary units. (C) Maximum actin polymerization rates were calculated from curves shown in panel B. (D) Times to half-maximal polymer were calculated from curves shown in panel B. (E) Actin polymerization assays using 2μM actin, 20nM Arp2/3 complex, and a combination of MBP, MBP-WHIMP(WCA), and MBP-WAVE2(WCA) are shown. AU, arbitrary units. (TIFF) [file pgen.1008694.s002.tiff]

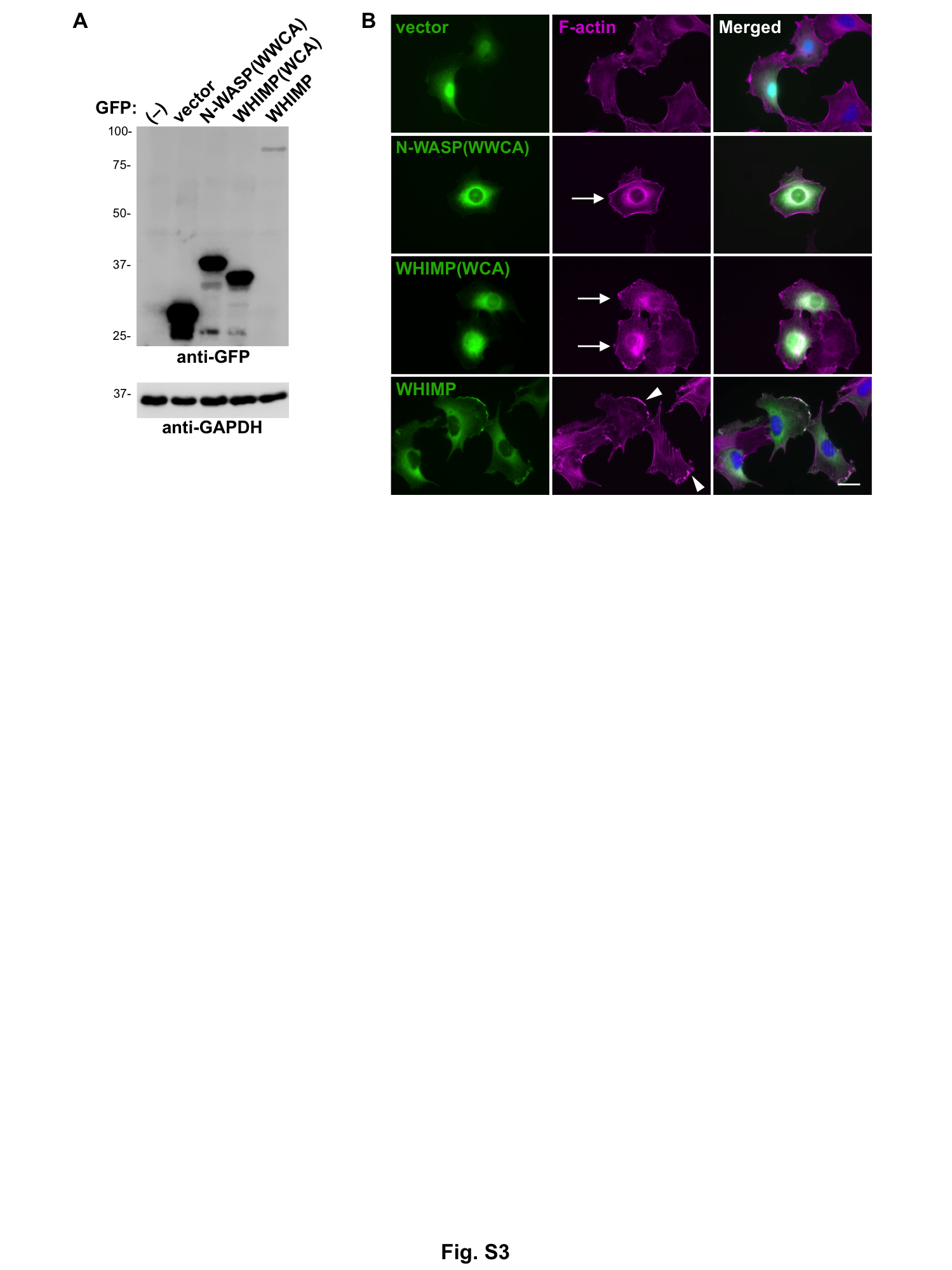

Supplement: S3 Fig — (A) B16-F1 cells transiently transfected with plasmids encoding GFP or GFP-tagged N-WASP(WWCA), WHIMP(WCA), or full-length WHIMP were lysed and subjected to SDS-PAGE and immunoblotting with antibodies to GFP and GAPDH. (B) Cells treated as in A were fixed and stained with phalloidin to visualize F-actin (magenta) and DAPI to label DNA (blue). Arrows highlight GFP-WCA-expressing cells with increased cytoplasmic F-actin content, while arrowheads indicate prominent F-actin-rich protrusions in GFP-WHIMP-expressing cells. Scale bar, 20μm. (TIFF) [file pgen.1008694.s003.tiff]

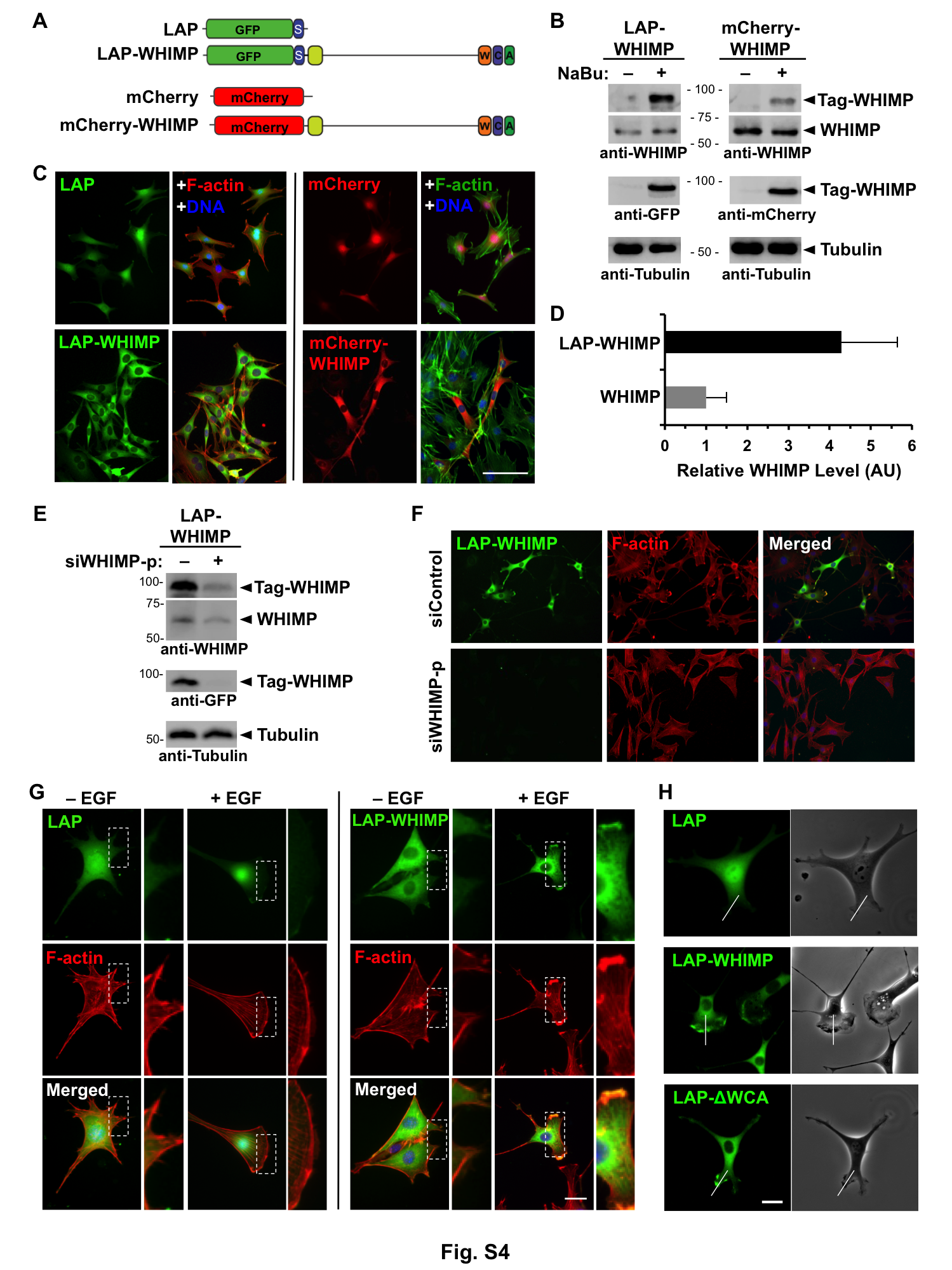

Supplement: S4 Fig — (A) Diagrams of the fusion proteins used to generate fluorescent NIH3T3 cell lines are shown. The LAP-tag includes GFP and an S-peptide. (B) NIH3T3 cells stably encoding LAP-WHIMP or mCherry-WHIMP were treated with 10mM sodium butyrate (NaBu) for 16-18h and sorted into GFP- or mCherry-expressing populations using flow cytometry. Following outgrowth, cells were left untreated (–) or were treated (+) with NaBu, lysed, and subjected to SDS-PAGE and immunoblotting with antibodies to WHIMP, GFP, mCherry, or Tubulin. The tagged and endogenous versions of WHIMP are indicated. (C) Cells treated as in B were fixed and stained with phalloidin to visualize F-actin and DAPI to label DNA. Scale bar, 100μm. (D) Cells induced to express LAP-WHIMP were immunoblotted with antibodies to WHIMP. The mean level of LAP-WHIMP relative to endogenous WHIMP was determined following densitometry of 6 representative blots +/-SD. (E) Cells induced to express LAP-WHIMP were treated with either a control siRNA or a pool of WHIMP-specific siRNAs for 48h, lysed, and subjected to SDS-PAGE and immunoblotting for WHIMP, GFP, and Tubulin. (F) Cells treated as in E were fixed and stained with phalloidin to visualize F-actin and with DAPI to label DNA. (G) Cells stably encoding LAP or LAP-WHIMP were induced, serum-starved, and stimulated with EGF for 5min. Cells were then fixed and stained with phalloidin and DAPI. Magnifications highlight LAP-WHIMP-specific enrichment at the cell periphery and co-localization with F-actin. Scale bar, 20μm. (H) Cells stably encoding LAP, LAP-WHIMP, or LAP-WHIMP(ΔWCA) were induced and examined live for GFP fluorescence and by phase-contrast microscopy. White lines indicate positions in which kymograph analyses were performed in Fig 5I. Scale bar, 20μm. (TIFF) [file pgen.1008694.s004.tiff]

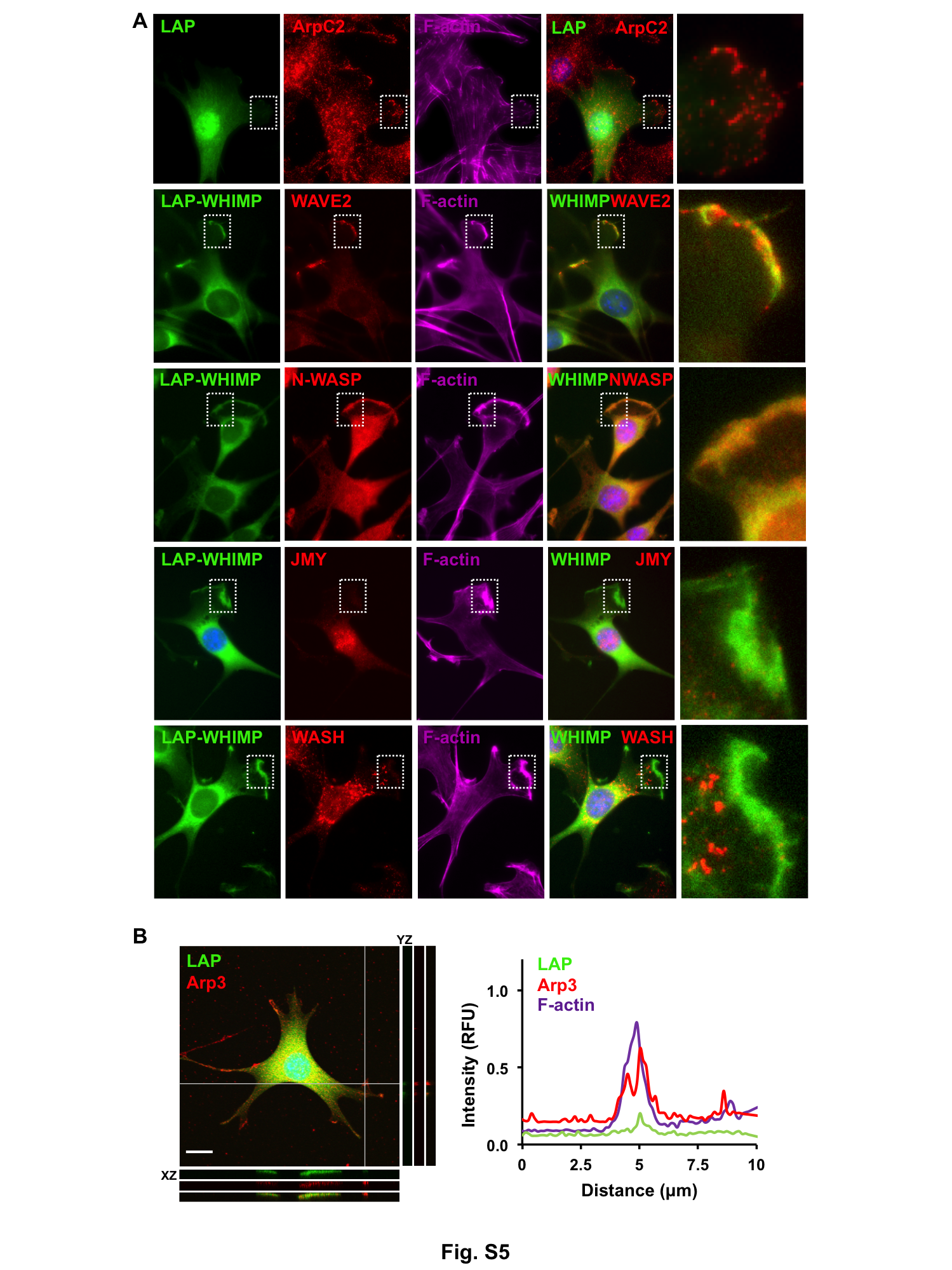

Supplement: S5 Fig — (A) NIH3T3 cell lines expressing a LAP tag or LAP-WHIMP were fixed and stained with antibodies to ArpC2, WAVE2, N-WASP, JMY, or WASH (red), phalloidin to visualize F-actin (magenta), and DAPI to label DNA (blue). Magnifications (right column) highlight LAP-WHIMP-specific localization to ruffles and overlap with WAVE2 and N-WASP. (B) LAP-expressing cells stained as in A were subjected to confocal microscopy for comparison to Fig 4D. Maximum intensity projections are shown in the large panel, and adjacent orthogonal views of YZ and XZ planes are indicated by the two gray lines. The plot profiles depict pixel intensities of LAP, Arp3, and F-actin along a 10μm line drawn near the intersection of the two gray lines. Scale bars, 20μm. (TIFF) [file pgen.1008694.s005.tiff]

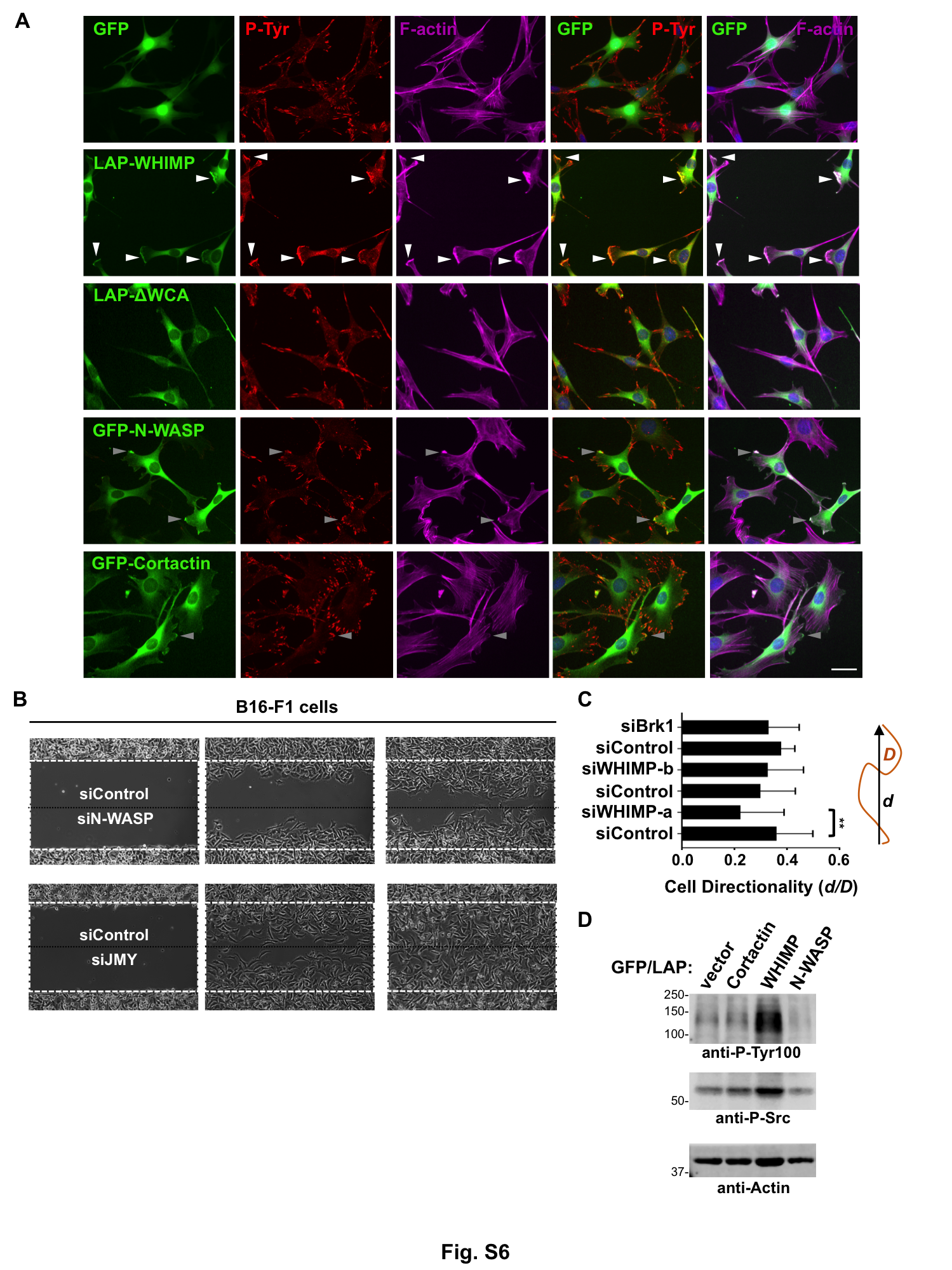

Supplement: S6 Fig — (A) NIH3T3 cells stably encoding GFP, LAP-WHIMP, LAP-WHIMP(ΔWCA), GFP-N-WASP, or GFP-Cortactin were treated with sodium butyrate for 16-18h, fixed, and stained with antibodies to detect phosphotyrosines (red), phalloidin to visualize F-actin (magenta), and DAPI to label DNA (blue). White arrowheads highlight large WHIMP- and F-actin-associated membrane ruffles, while grey arrowheads point to occasional small protrusions in N-WASP- or Cortactin-overexpressing cells. Scale bar, 20μm. (B) B16-F1 cells were treated with siRNAs, grown in cell reservoirs separated by a 0.5mm barrier, and analyzed by phase-contrast microscopy at 0h, 6h, and 12h after removing the barrier. Control cells are shown in the top chamber, and nucleation factor-depleted cells are shown in the bottom. The white dashed box encompasses the wound area at t = 0, and the black dashed line indicates the midpoint of the cell-free area. (C) A directionality index was calculated as the ratio of the distance between the starting and ending point “d” and the trajectory “D” based on the cell tracks described in Fig 8E. The bars represent the mean directionality +/-SD of cells (n≥20) for each siRNA treatment. **p<0.01 (two-tailed t-test). (D) Cells induced to express GFP, GFP-Cortactin, LAP-WHIMP, or GFP-N-WASP were subjected to SDS-PAGE and immunoblotting with antibodies to phosphotyrosine, active Src P-Tyr416 (P-Src), and actin. (TIFF) [file pgen.1008694.s006.tiff]

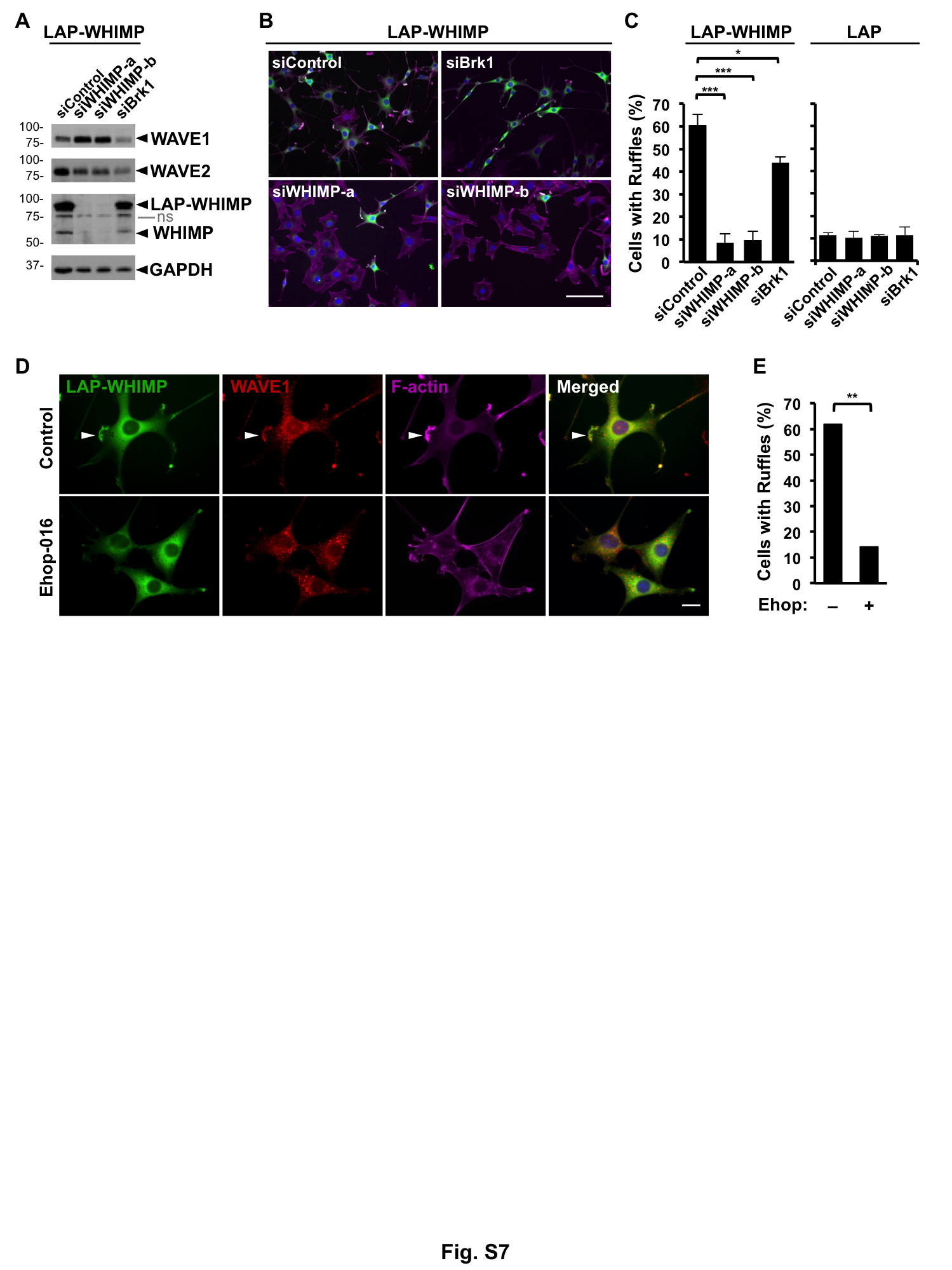

Supplement: S7 Fig — (A) NIH3T3 cells stably-encoding LAP-WHIMP were treated with a control siRNA, independent siRNAs to WHIMP, or an siRNA to Brk1, induced to express the fusion protein, lysed, subjected to SDS-PAGE, and immunoblotted with antibodies to WAVE1, WAVE2, WHIMP, and GAPDH. The tagged and endogenous versions of WHIMP are indicated. ns, non-specific. (B) LAP-WHIMP cells were treated with siRNAs, fixed, and stained with phalloidin (magenta), and DAPI (blue). Scale bar, 100μm. (C) The % of cells with ruffles was quantified. Each bar represents the mean % of cells (n = 200–300 per condition) with ruffles from 2–3 experiments +/-SD. *** p < 0.001, * p < 0.05 (ANOVA with Tukey post-test). Note that the baseline ruffling frequency in LAP control cells is approximately 10%, irrespective of siRNA-mediated targeting of endogenous WHIMP or Brk1. (D) LAP-WHIMP-expressing cells were treated with 4μM EHop-016 for 5h, fixed, and stained with an antibody to WAVE1 (red), phalloidin (magenta), and DAPI (blue). The arrowhead highlights the position of a prominent WHIMP-associated ruffle. Scale bar, 20μm. (E) Each bar represents the % of cells (n = 105–150 per condition) with ruffles from experiments performed in part D. ** p<0.01 (Fisher’s exact test). (TIFF) [file pgen.1008694.s007.tiff]

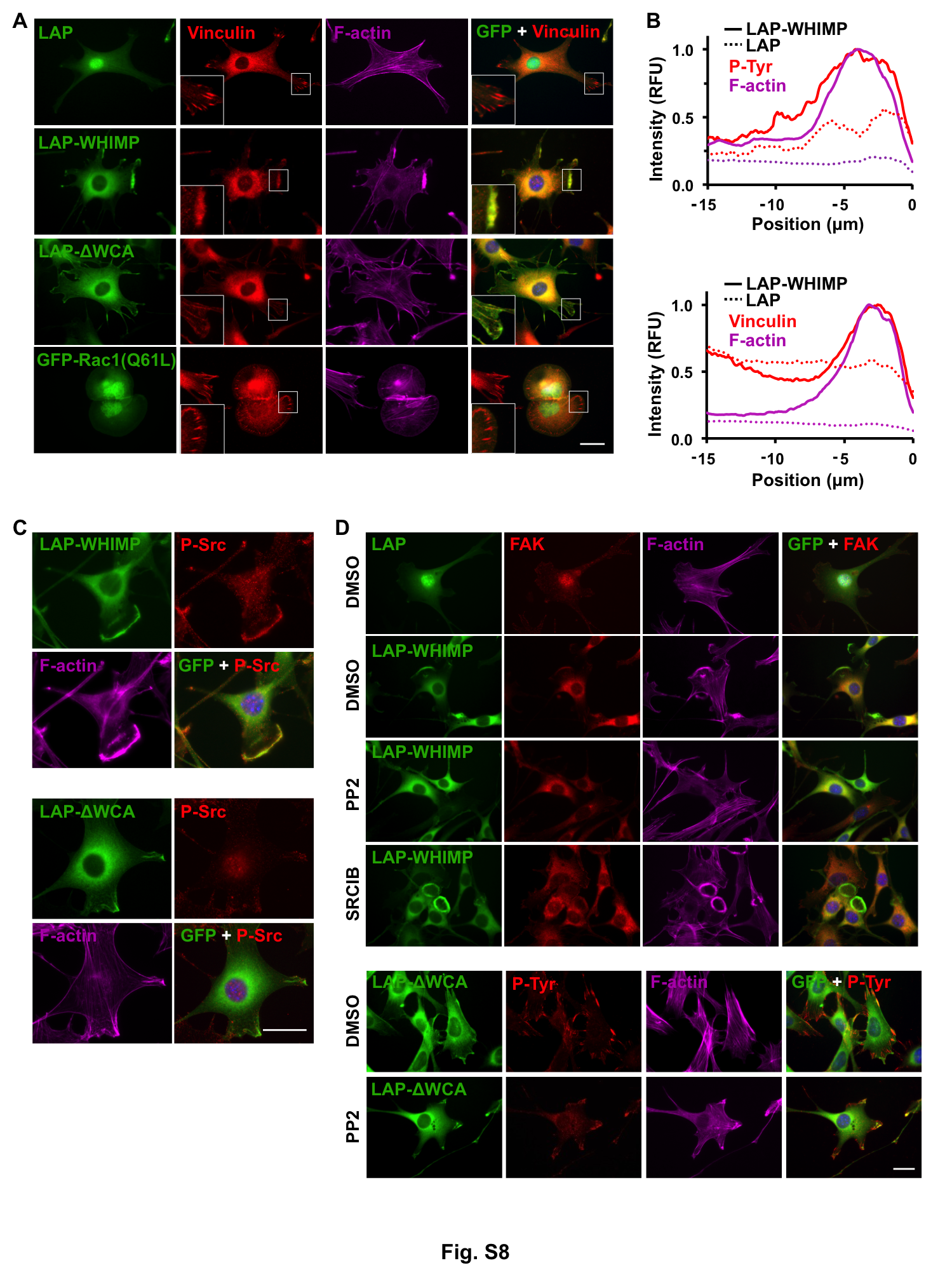

Supplement: S8 Fig — (A) NIH3T3 cell lines expressing LAP, LAP-WHIMP, or LAP-WHIMP(ΔWCA), or transiently transfected with a plasmid encoding GFP-Rac1(Q61L) were fixed and stained with antibodies to vinculin (red), phalloidin (magenta), and DAPI (blue). Insets highlight LAP-WHIMP localization at ruffles and broad enrichment of vinculin staining compared to wedge-like vinculin staining in other cells. (B) Line-scan plots depict the mean pixel intensity of phosphotyrosine (e.g., from Fig 9A), vinculin, and F-actin staining along 15μm lines near the edges of LAP and LAP-WHIMP cells (n = 10). The edges of the cells were set to 0μm on the X-axes. (C) Cell lines expressing LAP-WHIMP or LAP-WHIMP(ΔWCA) were fixed and stained with antibodies to active Src P-Tyr416 (P-Src; red), phalloidin (magenta), and DAPI (blue). (D) Cell lines expressing LAP, LAP-WHIMP, or LAP-WHIMP(ΔWCA) were treated with DMSO or with 20μM PP2 or Saracatinib (SRCIB) for 2h, fixed, and stained with antibodies to FAK or phosphotyrosine (red), phalloidin (magenta), and DAPI (blue). Scale bars, 20μm. (TIFF) [file pgen.1008694.s008.tiff]

**Fig.1D**

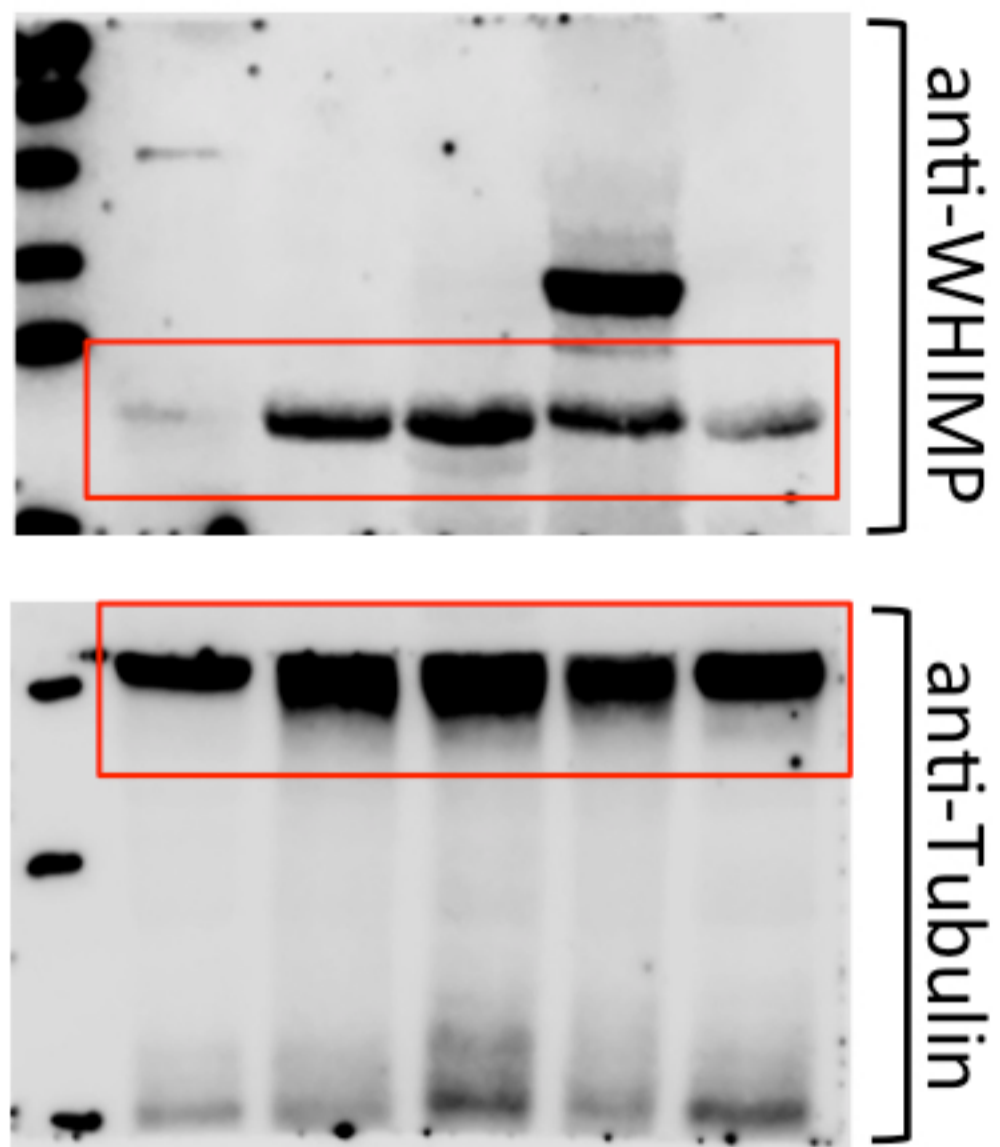

**Fig.1F**

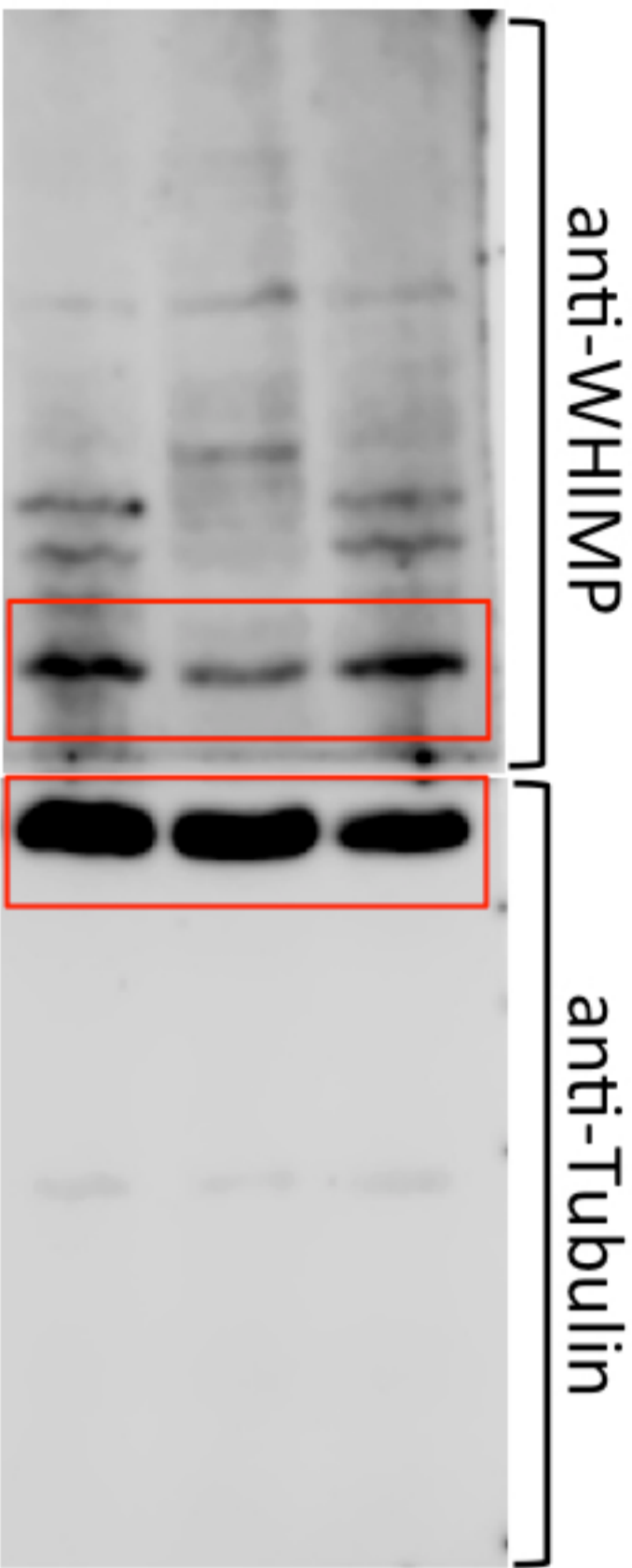

**Fig.1G**

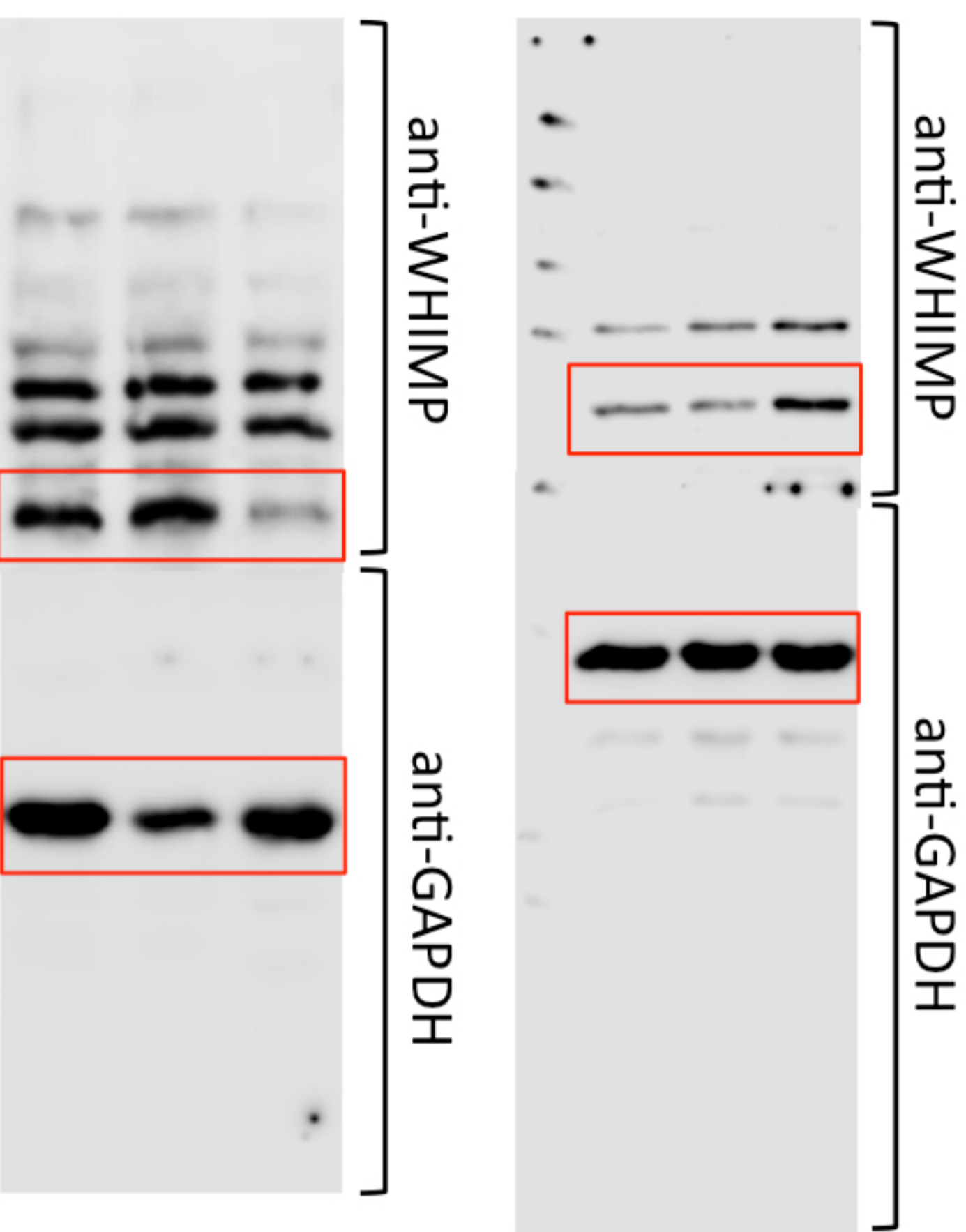

**Fig.3A**

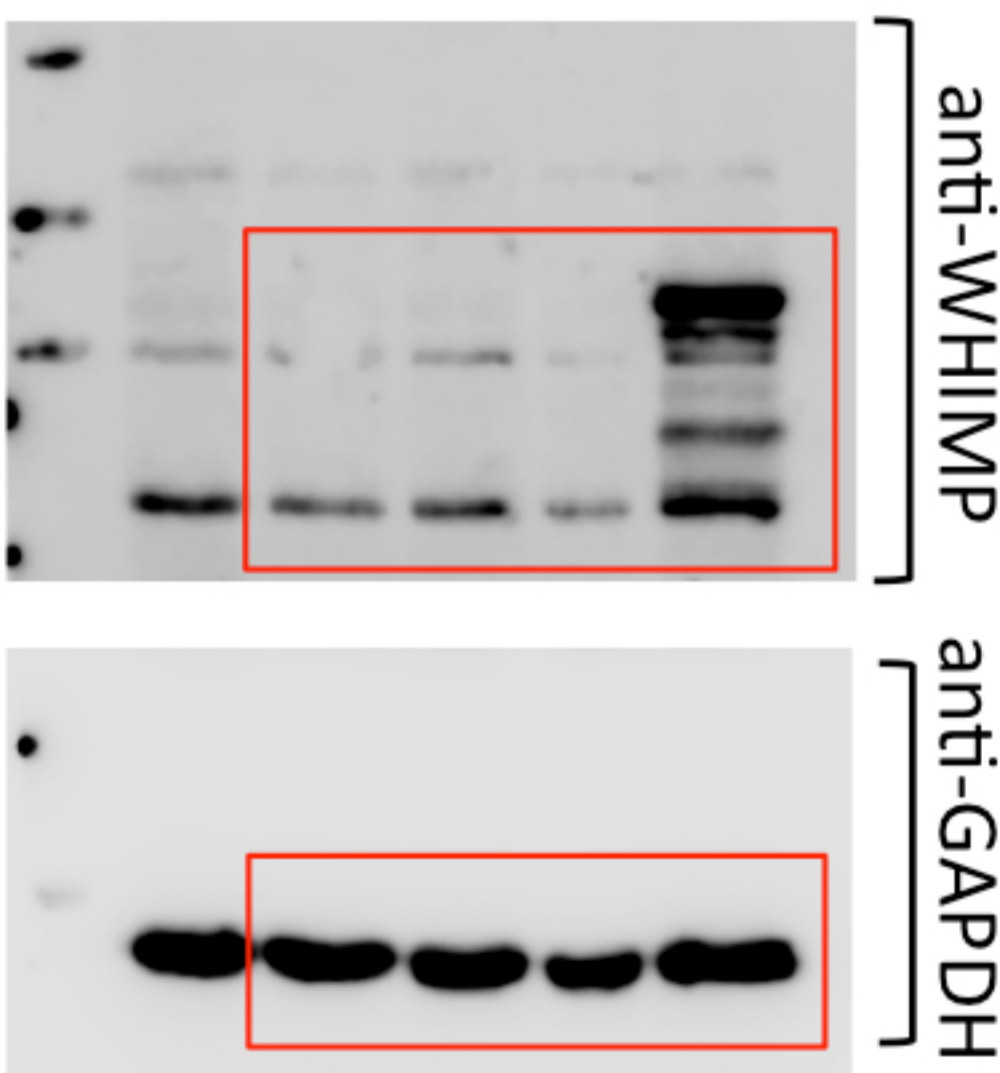

**Fig.5D**

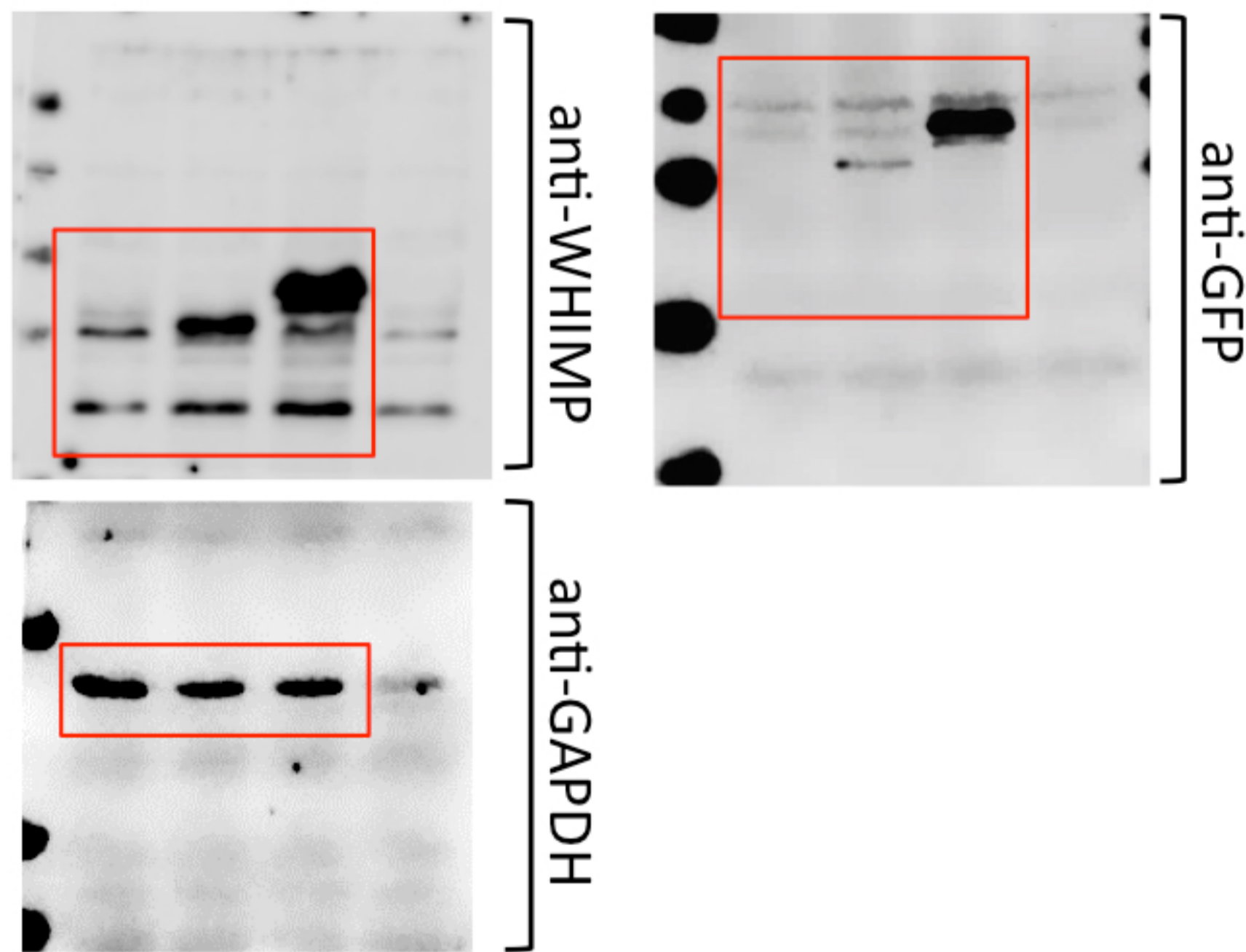

Fig.7A

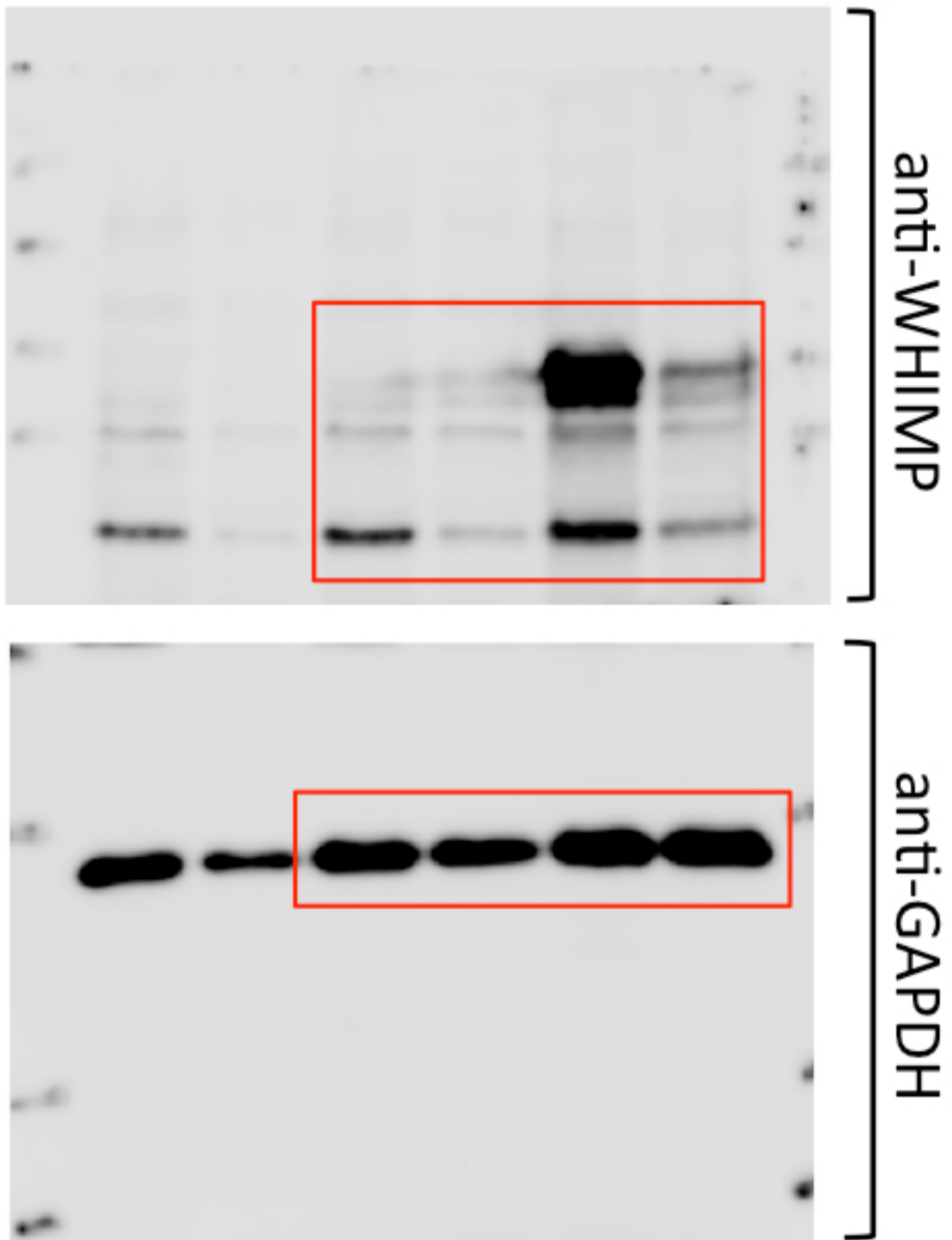

Fig.8A

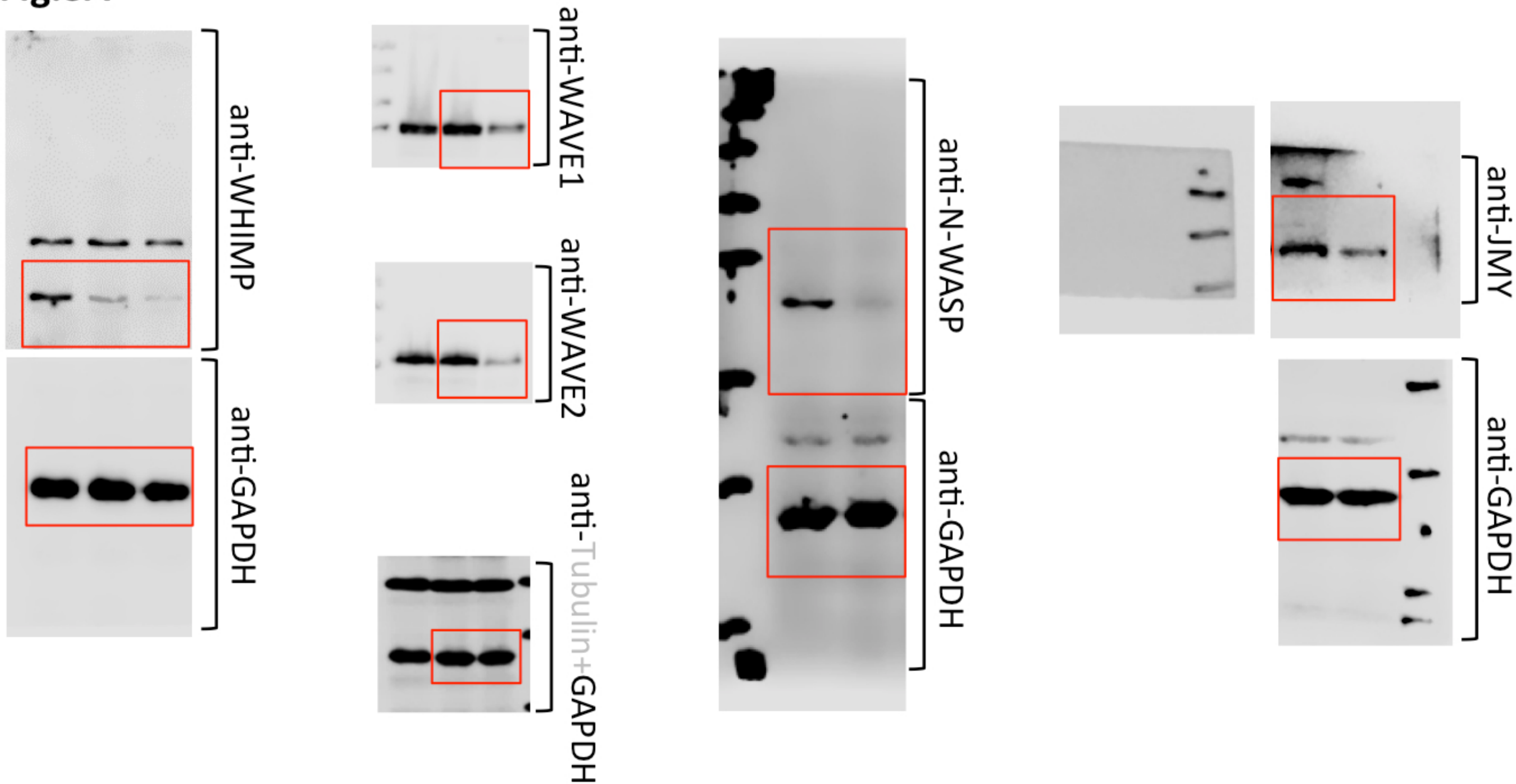

Fig. S9 (part 2)

Fig.9C

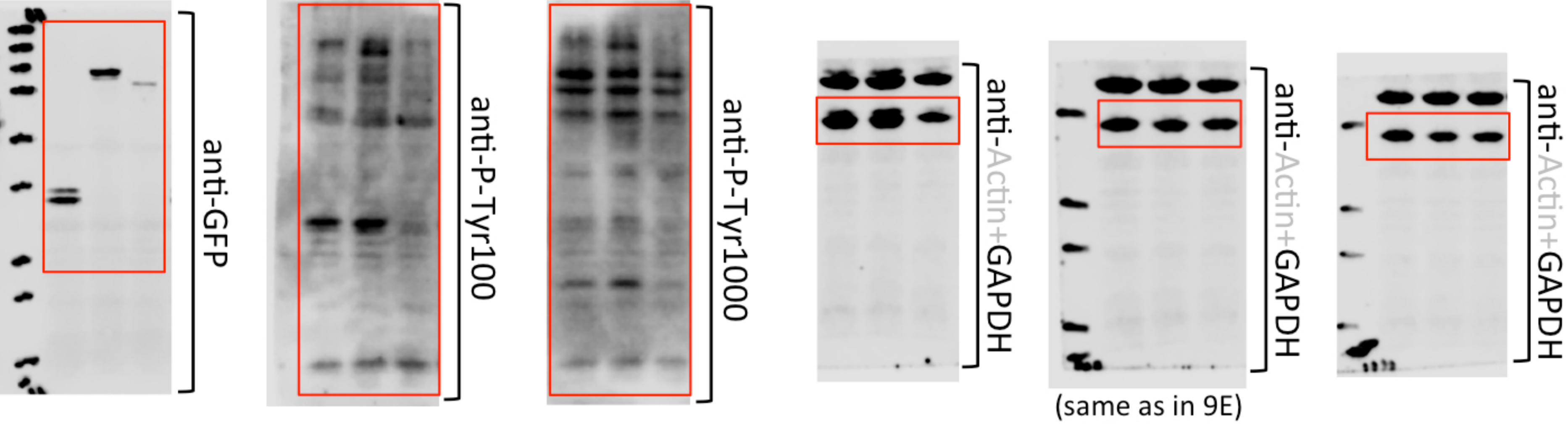

Fig.9E

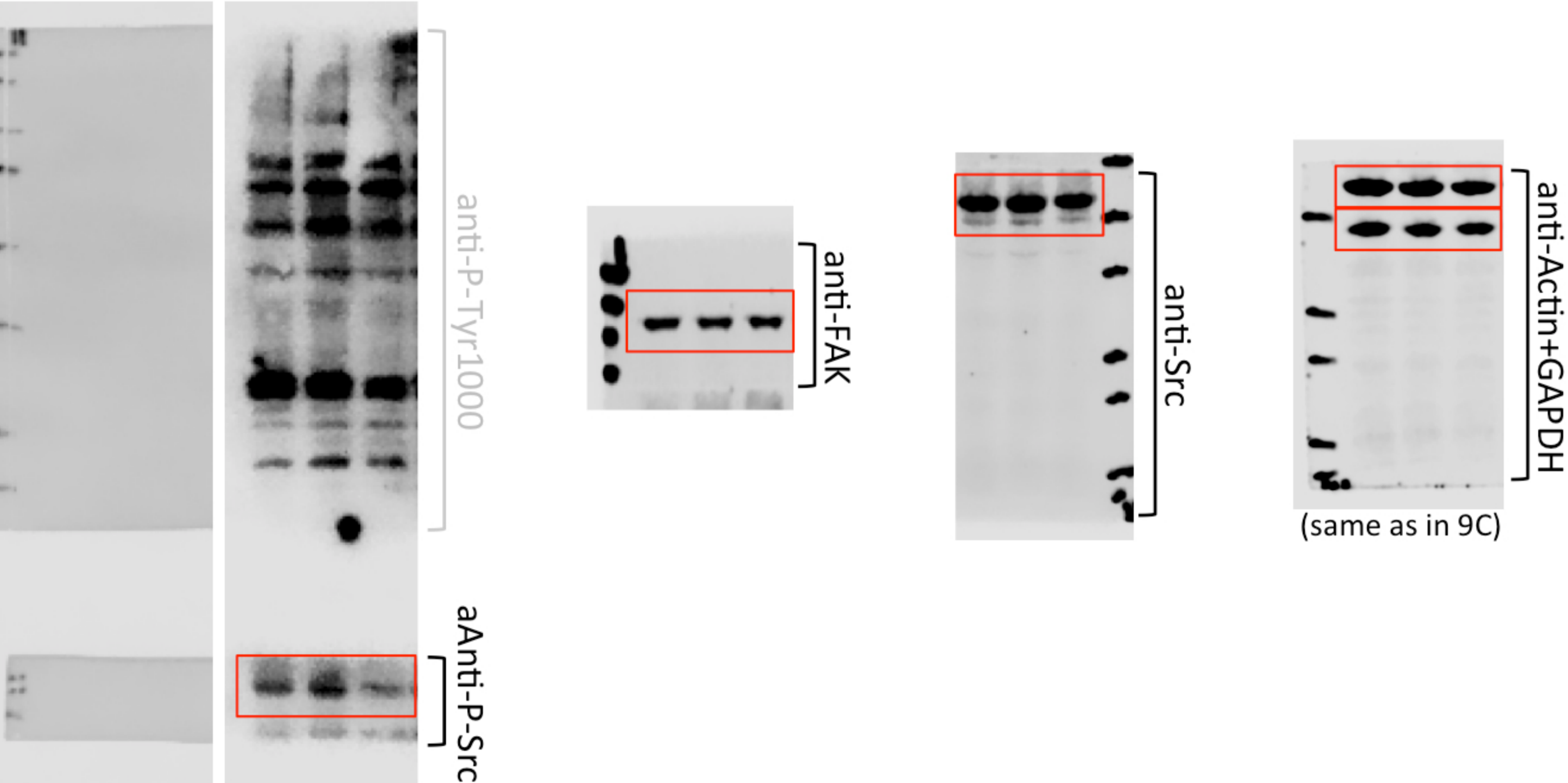

Fig.9F

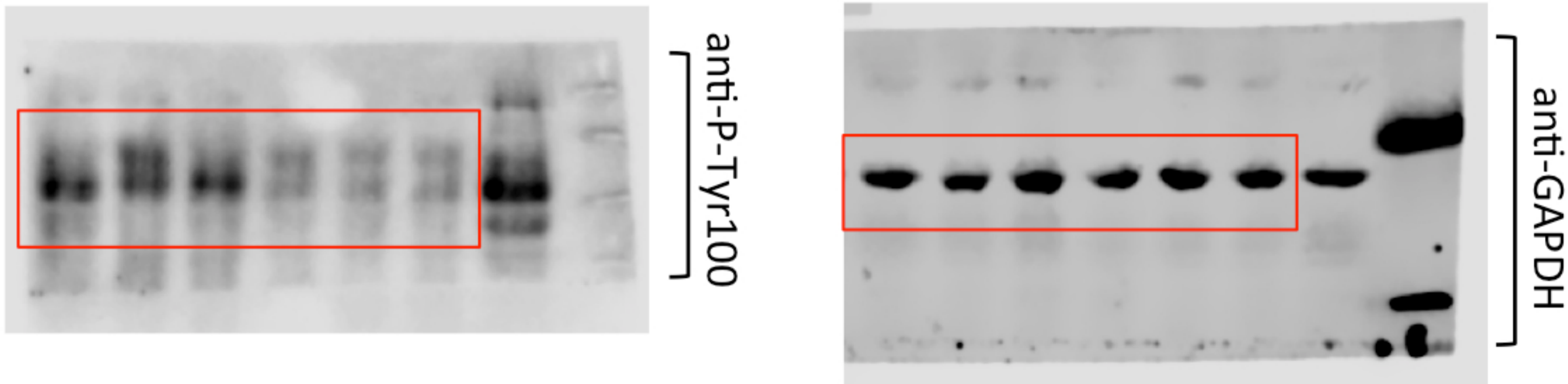

Supplement: S9 Fig — Post-cropped regions that are shown in Figs 1–10 are outlined with red boxes. (PDF) [file pgen.1008694.s009.pdf]
