# Supplementary material for: WHIMP links the actin nucleation machinery to Src-family kinase signaling during protrusion and motility
Source: PLoS Genet. 2020 Mar 20;16(3):e1008694. doi: 10.1371/journal.pgen.1008694 (PMC7112243; doi:10.1371/journal.pgen.1008694)
Supplement: S1 Table — (PDF) [file pgen.1008694.s011.pdf]

Description	Vector	Species	AA	R.E. Sites	Source
pKC-FastBac-MBP	pKC-FastBacMBP	N/A	N/A	N/A	Shen et al., 2012
pKC-FastBac-MBP-WHAMM	pKC-FastBacMBP	Human	1-809	KpnI-NotI	Shen et al., 2012
pKC-FastBac-MBP-WHIMP	pKC-FastBacMBP	Mouse	1-516	XhoI-HindIII	This study
pKC-FastBac-MBP-WASP(WCA)	pKC-FastBacMBP	Human	405-502	KpnI-NotI	This study
pKC-FastBac-MBP-N-WASP(WWCA)	pKC-FastBacMBP	Rat	386-501	KpnI-NotI	This study
pKC-FastBac-MBP-WAVE2(WCA)	pKC-FastBacMBP	Mouse	402-497	KpnI-NotI	This study
pKC-FastBac-MBP-WASH(WCA)	pKC-FastBacMBP	Human	326-465	KpnI-NotI	This study
pKC-FastBac-MBP-WHAMM(WWCA)	pKC-FastBacMBP	Human	659-809	KpnI-NotI	Shen et al., 2012
pKC-FastBac-MBP-JMY(WWWCA)	pKC-FastBacMBP	Mouse	817-983	KpnI-NotI	This study
pKC-FastBac-MBP-WHIMP(WCA)	pKC-FastBacMBP	Mouse	449-516	KpnI-NotI	This study
pKC-EGFP-C1 (vector)	pKC-EGFP-C1	N/A	N/A	N/A	Campellone et al., 2008
pKC-EGFP-N-WASP(WWCA)	pKC-EGFP-C1	Rat	386-501	KpnI-EcoRI	This study
pKC-EGFP-WHIMP(WCA)	pKC-EGFP-C1	Mouse	449-516	KpnI-NotI	This study
pKC-EGFP-WHIMP	pKC-EGFP-C1	Mouse	1-516	EcoRI-NotI	This study
pKC-mCherry-C1 (vector)	pKC-mCherry-C1	N/A	N/A	N/A	Campellone et al., 2008
pKC-mCherry-WHIMP	pKC-mCherry-C1	Mouse	1-516	EcoRI-NotI	This study
pKC-LAP-C1 (vector)	pKC-LAP-C1	N/A	N/A	N/A	Campellone et al., 2008
pKC-LAP-WHIMP	pKC-LAP-C1	Mouse	1-516	SpeI-EcoRI	This study
pKC-LAP-WHIMP(ΔWCA)	pKC-LAP-C1	Mouse	1-448	SpeI-EcoRI	This study
pGFP (pKC425) (vector)	pCDNA3::GFP	N/A	N/A	N/A	Campellone et al., 2008
pGFP-N-WASP	pCDNA3::GFP-Flag	Rat	1-501	KpnI-EcoRI	Campellone et al., 2008
pGFP-Cortactin	pCDNA3::GFP	Mouse	1-546	EcoRI-BamHI	Campellone et al., 2008
pmCherry-Rab5a	pmCherry-C1	Mouse			Addgene #27679
pmCherry-Rab5a(Q79L)	pmCherry-C1	Human			Addgene #35138
pEGFP-Rac1(Q61L)	pCDNA3-EGFP	Human			Addgene #12981

Table S1
