# Supplementary material for: WHIMP links the actin nucleation machinery to Src-family kinase signaling during protrusion and motility
Source: PLoS Genet. 2020 Mar 20;16(3):e1008694. doi: 10.1371/journal.pgen.1008694 (PMC7112243; doi:10.1371/journal.pgen.1008694)
Supplement: Attachment — Submitted filename: KabrawalaPLOScoverletter2020.pdf [file pgen.1008694.s015.pdf]

UCONN
UNIVERSITY OF CONNECTICUT

DEPARTMENT OF MOLECULAR AND CELL BIOLOGY
INSTITUTE FOR SYSTEMS GENOMICS

KENNETH G CAMPELLONE, PHD
ASSOCIATE PROFESSOR
67 NORTH EAGLEVILLE RD - RM 216
STORRS, CT 06269-3197

PHONE: (860) 486-3326 LAB: (860) 486-2497
EMAIL: KENNETH.CAMPELLONE@UCONN.EDU
WEB: HTTPS://CAMPELLONELAB.UCONN.EDU
TWITTER: @CAMPELLONELAB

February 8, 2020

Dear *PLOS Genetics* Editors,

Thank you for your evaluation of our paper entitled "***WHIMP links the actin nucleation machinery to Src-family kinase signaling during protrusion and motility***". We appreciate the reviewers' constructive comments. Based on their critiques, we are submitting a revised version here. As you will see, we have moved a supplemental figure to the main paper, added more data to the supporting information, and clarified a few results/conclusions in the text. Our point-by-point responses to the reviewers' specific comments are attached below.

Thank you for considering our revised manuscript for publication.

Sincerely,

Ken Campellone

kenneth.campellone@uconn.edu

Dear Dr Campellone,

Thank you very much for submitting your Research Article entitled 'WHIMP links the actin nucleation machinery to Src-family kinase signaling during protrusion and motility' to PLOS Genetics. Your manuscript was fully evaluated at the editorial level and by independent peer reviewers. The reviewers appreciated the attention to an important topic but identified some aspects of the manuscript that should be improved.

In addition to suggested revisions to the text of the manuscript by all three reviewers, Reviewer 1 also suggests a few additional analyses that may strengthen the paper. Additionally, Reviewer 3 also makes the reasonable requests that full images of the Western Blots be included as supplemental data.
--As described below, we have clarified several results in throughout the text and supplemental sections. We have also added to Fig.S7C to show that we do not detect differences in ruffle numbers upon deletion of WHIMP or the WAVEs in the weakly-protrusive NIH3T3 cell line. These results further validate our use of NIH3T3 cells primarily for gain-of-function studies and B16-F1 cells mainly for loss-of-function studies. At the request of one of the reviewers, we have moved one of the supporting figures to the main paper as a new Fig.6. Finally, we have added images showing the paper's pre-cropped blots as a new 3-page Fig.S9.

Although the reviewers did not comment on this point, we would also ask you to clarify the controls for your western blots in Figures 8c and 8e; the GAPDH bands in these two panels are very similar. If they are indeed the same image, this should be called out explicitly in the figure legend and the same done for any other reused panel items.
-- The GAPDH blots in 8C and 8E (now 9C and 9E) are the same - they were from the same samples and experiment, which we now state in the main figure legend and depict in Fig.S9.

We ask you to modify the manuscript according to the review recommendations before we can consider your manuscript for acceptance. Your revisions should address the specific points made by each reviewer. In addition we ask that you: 1) Provide a detailed list of your responses to the review comments and a description of the changes you have made in the manuscript. 2) Upload a Striking Image with a corresponding caption to accompany your manuscript if one is available (either a new image or an existing one from within your manuscript). If this image is judged to be suitable, it may be featured on our website. Images should ideally be high resolution, eye-catching, single panel square images. For examples, please browse our archive. If your image is from someone other than yourself, please ensure that the artist has read and agreed to the terms and conditions of the Creative Commons Attribution License. Note: we cannot publish copyrighted images.
--The detailed list of our responses to the reviewer comments appear below. We have also uploaded a Striking Image.

We hope to receive your revised manuscript within the next 30 days. If you anticipate any delay in its return, we would ask you to let us know the expected resubmission date by email to plosgenetics@plos.org.

If present, accompanying reviewer attachments should be included with this email; please notify the journal office if any appear to be missing. They will also be available for download from the link below. You can use this link to log into the system when you are ready to submit a revised version, having first consulted our Submission Checklist.

While revising your submission, please upload your figure files to the Preflight Analysis and Conversion Engine (PACE) digital diagnostic tool. PACE helps ensure that figures meet PLOS requirements. To use PACE, you must first register as a user. Then, login and navigate to the UPLOAD tab, where you will find detailed instructions on how to use the tool. If you encounter any issues or have any questions when using PACE, please email us at figures@plos.org.

Please be aware that our data availability policy requires that all numerical data underlying graphs or summary statistics are included with the submission, and you will need to provide this upon resubmission if not already present. In addition, we do not permit the inclusion of phrases such as "data not shown" or "unpublished results" in manuscripts. All points should be backed up by data provided with the submission.

PLOS has incorporated Similarity Check, powered by iThenticate, into its journal-wide submission system in order to screen submitted content for originality before publication. Each PLOS journal undertakes screening on a proportion of submitted articles. You will be contacted if needed following the screening process.

To resubmit, you will need to go to the link below and 'Revise Submission' in the 'Submissions Needing Revision' folder.

Please let us know if you have any questions while making these revisions.

Yours sincerely,
Lillian Fritz-Laylin, PhD Guest Editor PLOS Genetics
Gregory Barsh Editor-in-Chief PLOS Genetics

Comments to the Authors:

Reviewer #1: Summary The manuscript by Kabrawala et al reports a novel Nucleation Promoting Factor (NPF), which the authors have named WHIMP. The study utilizes bioinformatics, as well as biochemical and cell biological approaches to conclude that WHIMP drives membrane protrusion and cell migration by activating Src family kinases. The authors also conclude that a complex interplay exists between NPFs, as disruption of the WAVE complex impacts WHIMP-dependent membrane ruffling. The authors have conducted a robust, thorough study (as evidenced by the body of supplemental data and nine main figures). The finding that WHIMP is a bona fide NPF is significant enough in my mind to warrant interest from the field. Though WHIMP is absent from the human genome, novel NPFs may yet be discovered in light of the present findings. Additionally, mouse cell lines like NIH 3T3 and B16-F1 are used routinely to model cellular behavior, and as such it is critical to understand the complete complement of cytoskeletal regulators in these cells. The manuscript is generally well written and most of the authors' conclusions are well supported by the data. I support publication of this manuscript, but would like to hear the authors' views on the following points and to

provide a few pieces of key additional data, where appropriate.
--We thank this reviewer for being tough but fair.

Major points It is clear that the authors have responded to a number of critiques regarding this manuscript. The major outstanding concern that I have is that many of the conclusions are based on ectopic overexpression of WHIMP. It is clear that transfected cells demonstrate a number of robust phenotypes consistent with WHIMP's ability to promote dendritic actin nucleation at membranes, but the contribution of endogenous WHIMP to membrane protrusion and F-actin generation remains unclear.
1) Much of the paper is dedicated to understanding how GFP-WHIMP(WCA), mCherry-WHIMP, or LAP-WHIMP affect cellular protrusion and F-actin generation. Have the authors considered whether the overexpression of WHIMP above physiological levels might be inducing it to mis-localize to protrusions or become hyper-activated, leading to aberrant dorsal ruffle production? Are the authors able to stain endogenous WHIMP with their antibody?
--Yes, we've considered that overexpression of WHIMP might cause it to mislocalize or alter its activity (especially in light of the observation that the initial discovery of WASH erroneously indicated that it localized to filopodia and lamellipodia). Such caveats are inherent to the study of all WASP-family members, so we monitored the expression levels of tagged WHIMP pretty carefully throughout the paper, relied mostly on stably-transfected cells (which provide more consistency in expression levels), and even tested for exaggerated ruffling by making cell lines expressing tagged N-WASP or Cortactin. All of our gain-of-function (as well as loss-of-function studies described below) point to specific role for WHIMP in protrusion and motility. For staining with the anti-WHIMP antibody, unfortunately we observed a high level of background staining (everywhere) during our initial studies, and we haven't worked out conditions that work reliably for immunofluorescence yet.
Figure 5 demonstrates that NIH3T3 fibroblasts expressing endogenous levels of WHIMP don't ruffle nearly as much as when LAP-WHIMP has been expressed on top of endogenous WHIMP. A knockdown/rescue approach might work to address this point, as the authors could sort siRNA WHIMP cells re-expressing mCherry-WHIMP based on fluorescence intensity. The authors can identify a population of cells that expresses mCherry-WHIMP near endogenous levels. They can then better assess the localization of mCherry-WHIMP and its ability to induce dorsal ruffling and membrane protrusion relative to control and siWHIMP cells. This is a major point that in my mind warrants additional experimentation.
--As the reviewer notes, in Fig.5 (and in general), the NIH3T3 cells exhibit a low steady-state level of ruffling. We have added data to Fig.S7C showing that this basal ~10% of ruffling 3T3s is observed whether or not we treat with siRNAs targeting WHIMP or even Brk1 (for depleting the WAVEs). We don't have enough sensitivity to study a decrease in ruffling when ruffling is already inefficient. The limited amount of steady-state protrusion and slow speeds of ruffling by 3T3s is what caused us to carry out most of our loss-of function studies in the more protrusive and faster B16-F1 cells. In summary: RNAi-mediated depletion of endogenous WHIMP in NIH3T3 cells resulted in less wound closure (Fig.7B,E); RNAi-mediated depletion of endogenous WHIMP in B16-F1 cells resulted in less wound closure (Fig.8B-D), slower cell motility (Fig.8E-F), fewer/slower/smaller ruffles (Fig.8G-I), and less tyrosine phosphorylation (Fig.9F). The knockdown+rescue approach is clever suggestion, but has not been technically

achievable in our hands. Even when we have sorted "high" and "medium" fluorescent WHIMP expressers (and even performed secondary re-sorts), by the time the cells are ready for functional analyses, the most uniform batches of cells overexpress WHIMP by about 4-fold. We haven't been able to isolate populations of cells that consistently express tagged WHIMP at levels equal to endogenous WHIMP as we did previously for WHAMM [Ref.34]. The closest we could come to doing a knockdown+reexpression experiment was in the context of 3T3 wound closure in Fig.7, where knockdown of endogenous WHIMP resulted in slower closure, overexpression of tagged WHIMP resulted in faster closure, and knockdown/reexpression featuring similar levels of endogenous and tagged WHIMP resulted in closure that most closely resembled control endogenous conditions.

One brief note on Supplementary Figure 8: This data seems supportive of the authors' overall conclusions. siRNA knockdown of WHIMP decreases ruffling. However, the siCtl cells are overexpressing LAP-WHIMP. The authors should also include siCtl NIH3T3 cells expressing endogenous levels of WHIMP as well as siWHIMP cells. It may be that the percentage of cells with ruffles is not so different in siCtl NIH3T3 cells and WHIMP KD NIH3T3 cells.

--The reviewer is correct, and we have added the requested data. Depletion of endogenous WHIMP or endogenous Brk1 did not reduce ruffling below 10% in control 3T3 LAP cells (new Fig.S7C). Due to the lower basal activities of 3T3s, we focused our loss-of-function efforts mostly in B16-F1 cells. Depletions of endogenous WHIMP or Brk1 significantly reduced multiple ruffling parameters in the B16 cells (Fig.8G-I).

2) The authors should consider more thorough analysis of their siRNA WHIMP cells compared to siCtl NIH3T3 expressing endogenous WHIMP. Are membrane protrusions and/or dynamics, or dorsal ruffles compromised? Are F-actin levels altered? What about p-SFK and p-Tyr levels? The siRNA data in Figures 6 and 7 are quite nice and ultimately suggest that these additional investigations will likely be fruitful. The experiments proposed above may yield additional mechanistic insight into how WHIMP alters cell motility and cellular architecture. It appears the authors have conducted some of these studies with B16-F1 cells in Figure 7 and 8F, which supports their overall interpretation of WHIMP function. Conducting similar studies in NIH3T3 cells should therefore be fairly straightforward. It may also be worthwhile to report the relative difference in endogenous WHIMP expression in NIH3T3 versus B16-F1 cells at baseline. This may be interesting given that B16-F1 cells seem more motile and ruffle more than NIH3T3 cells at baseline (e.g. Figure 5B versus Figure 7G and Figure 6E versus Figures 7 C-D).

--Similar to point 1, yes we considered (and performed some) further analyses of 3T3s. As mentioned above, we added a supplemental panel showing that depletion of endogenous WHIMP did not reduce ruffling below 10% in control 3T3 cells (new Fig.S7C). Importantly, targeting Brk1 in order to deplete the WAVEs (established protrusive factors) also did not reduce this baseline level of ruffling. In light of these observations, we chose not to chase after phenotypes in 3T3 cells that don't provide a useful dynamic range for studying steady-state ruffling in our hands. Overall, experiments in 3T3 cells that ruffle inefficiently (Fig.5B,10C;S7C) have not been informative in our loss-of-function studies using siRNAs to WHIMP or Brk1, but they have been very informative in gain-of function studies involving protein overexpression. On the other hand, the B16 cells that normally ruffle at >80% frequency (Fig.8G) were quite amenable to performing loss-of-function studies using siRNAs to WHIMP or Brk1. Regarding F-

actin levels, we've studied knockdowns/knockouts of every WASP-family member over the years, and have never seen a decrease in total F-actin content; we only see changes in F-actin content (increases) in overexpression studies. Finally, we have not directly studied WHIMP expression side-by-side in 3T3s and B16s, this is because our separate immunoblotting results have never led us to believe that its expression is very different between the two cell lines. We can't attribute the differences in 3T3 vs B16 protrusion/motility to WHIMP expression – the numerous biological differences between these cell lines (immortalized embryonic fibroblasts vs skin cancer-derived epithelial-like cells) are likely to contribute to these phenotypes.

Minor points 1) The authors should consider editing their schematic diagram in Figure 9E. It is not clear at first glance what the blue arrows indicate to the reader.
--The blue arrows indicate the newly-discovered functions of WHIMP in Arp2/3 activation (direct interaction, solid line) and in promoting SFK activation (possibly indirect interaction, dashed line). Previously known pathways involving other factors are shown in black. We have clarified this in the legend.

2) It is sometimes unclear why the authors switch between NIH3T3 and B16-F1 cells. For example, Figure 8 is largely conducted with NIH3T3 cells, aside from panel F. Should the data be re-organized? Alternatively, a short rationale would be useful to the reader.
--As mentioned elsewhere, the limited amount of steady-state protrusion and slow speeds of 3T3s is what caused us to carry out most of our gain-of function studies in that cell background, while the more protrusive and faster B16-F1 cells were more amenable to most of our loss-of function studies. To clarify this more, we have added some additional text mentioning the advantage of studying protrusion and motility in B16-F1 cells in lines 388-390.

3) The authors conclude the abstract by making a statement about 'feedback control' of WASP-family proteins/SFKs. While their data is supportive of this idea, especially with respect to the p-Tyr signaling, one can imagine alternative possibilities. For Figure 7 (especially panel G): Perhaps WHIMP and WAVE1/2 are both required in parallel to initiate the dramatic ruffle-based protrusion. Figure 9C indicates that LAP-WHIMP cells still generate a significant seeming ruffle phenotype upon SFK inhibition. While SFK may activate (and be activated by) WHIMP, this seems to imply that WHIMP's activity is to some extent SFK-independent. Perhaps SFK can activate WHIMP and WAVE in parallel, but WHIMP is required to sustain SFK activity, which then acts as a positive feedback mechanism to sustain WAVE. A discussion of these points (and alternative organizations of the pathway) may fit well at the end of the paper (and inform the construction of Figure 9E, as mentioned above).
--We added some text to the Discussion about parallel WHIMP/WAVE activities and other tyrosine kinases. We don't like to be very speculative, but we think we have a basic description of several possibilities in there.

4) Overall the discussion section of the paper is well written. However, it reads like a lengthy recap of the results with a first paragraph that largely touches on already introduced information from the introduction. The authors may want to consider streamlining this section a bit more so that they can unpack the concept of NPF coordination and how WHIMP might

intersect with GTPase/SFK/WAVE signaling, especially given that there are many ways in which such regulation might work. I realize that much of this comes down to personal preference and style, so I would not consider this a requirement for publication.
--We truncated some earlier parts of the Discussion, but added text as suggested in point 3, for a net shortening of 3 lines.

Reviewer #2: In the manuscript entitled "WHIMP links the actin nucleation machinery to Src-family kinase signaling during protrusion and motility" Kabrawala et al. discover a new actin nucleation promoting factor encoded on the X-chromosome, WHIMP. WHIMP is considered a new WASP family member and using bulk assays the authors show that it promotes actin assembly by activating the Arp2/3 complex in fluorescence-based bulk actin assembly assays. Using several mammalian cell lines the authors show that the presence of more WHIMP in cells promotes actin assembly behaviors mediated by the Arp2/3 complex (i.e. cell ruffling, migration, wound healing), and that less WHIMP protein results in reduced motility and protrusion. Using pharmacological agents, the authors show that WHIMP also activates cellular tyrosine kinase signaling cascades. This work addresses all the experiments this reviewer can think of as required to characterize a new Arp2/3 complex nucleation promotion factor as well as probes intriguing signaling / cell regulatory mechanisms. Congratulations to the authors to this work.
--Thanks.

Below are specific concerns and points of clarification:

- Overall the introduction reads long, but there is value in explaining all the caveats of the WASP family proteins.

--We agree, and wanted to ensure that a thorough summation of the WASP-family field was presented. We have shaved a few lines off this section and added a line as requested below. The Intro is 2 pages + 8 lines (double-spaced) in length, which seems reasonable.

- Referring to WASP and Cortactin as "nucleation factors" is confusing. Neither is a bona fide actin nucleation protein (i.e. the Arp2/3 complex, formins, etc), but rather "nucleation promoting factors" which indirectly stimulate actin assembly by activating the canonical nucleation protein.

--We explicitly use the term "nucleator" to describe true nucleators like the Arp2/3 complex and Formins, and are careful to not describe WASP-family proteins as "nucleators" (although JMY would be an exception). We use "nucleation-promoting factor" or (depending on the context) "nucleation factor" to describe the WASP-family proteins. We think that this nomenclature is clearer in the revised manuscript.

- A nice addition/point to make around line 64/65 could be mentioning the balance between Arp2/3 and formin (mDia2) activities by Dip/Wish/Spin90.

--As suggested, we have expanded this sentence to mention other Dip-interacting actin nucleators and nucleation-promoting factors.

• The authors should consider reordering the sequences found in Figures 1A and 1B. For reader clarity, it might be easier to make the comparison of WHIMP to WASP if WHIMP was on the first line rather than the last line. Or possibly reorder to make the WHIMP sequence closer to WAVE1 since the sequence homology is highest on the amino acid level?
--We thought about this, but given the generally limited homology between WHIMP and all other factors, and the summary schematic in panel C, we prefer to keep them in chronological order of discovery in A-B.

• The data presented in Figure 2B imply that WHIMP cannot nucleate actin assembly on its own. I am confused by wording of other information in Figure 2S which implies WHIMP can nucleate actin alone, but only at high concentrations. Noting the concentration of WHIMP that is expected to be closest to the physiological concentration might clarify this finding for readers.
--Fig.2B and Fig.S2B both demonstrate that WHIMP-mediated actin assembly is dependent on the Arp2/3 complex at all of the concentrations that we tested. We have double-checked the text to make sure that we do not imply otherwise. The concentrations that we used in pyrene-actin polymerization assays (from low up to saturation) are consistent with those shown elsewhere in the literature. As with most (all?) mammalian nucleators or nucleation-promoting factors, the actual concentration of WHIMP in cells has not been quantitatively measured.

• The authors should clarify further whether WHIMP is actually capable of binding profilin-actin or if this is an assumption due to the lack of PLP peptides (lines 133-134).
--As mentioned in the text, WHIMP lacks a polyproline peptide, so it is not predicted to bind profilin, at least not in the same way that other WASP-family members do. We don't dwell on this observation and don't know if it binds profilin via other sequences, as we lack reagents to experimentally test this possibility.

• Can the authors please comment on whether the reduced protein and gene expression of WHIMP in Figures 1D and 1E is because it is on the X chromosome? The N2A and HT22 cells used are derived from male mice, so they effectively have one less dose of X-chromosome? Perhaps information can be determined or tracked from the population of mice used in 1D?
--That is an interesting question. The answer is that we don't know (but don't think so). Determining the actual degree of X-chromosome dosage compensation or WHIMP expression during development is beyond our capacity at this point.

• How many cells did GFP-WHIMP localize to cell peripheral membranes (lines 216-217). Was very few or were they easy to find?
--Whenever the 3T3 cells had F-actin-rich ruffles, the ruffles contained GFP-WHIMP (i.e., easy to find). We have clarified the consistency of WHIMP localization to protrusions in the text and describe it in greater detail in subsequent sections of the paper.

• Figure 3C the significant increase in actin intensity with GFP-WHIMP constructs doesn't actually look like it is 25%... are these analyses done in a blind manner? The authors should clarify and also add some text about their positive control (N-WASP).

--Experiments are generally performed in a blinded manner (i.e, using numbered coverslips), although when cells have ruffles, the observer can tell which cells are expressing full-length WHIMP, because the other GFP-fusions don't localize to the ruffles. In 3C, across the 25 cells quantified for each construct, the figure shows means of 1.00, 2.07, 1.20, and 1.25. The 2-fold increase in F-actin content in N-WASP(WWCA)-expressing cells is mentioned in lines 218-219. Additional F-actin quantifications for B16-F1 cells are found in Fig.5F.

• There is a significant amount of text devoted to the WHIMP micropinocytosis assay, however all this data is placed in the supplemental file. Either this text should be reduced/bottom-lined or Figure S7 should be introduced into the main text. The finding/dissection of the role of WHIMP in nonspecific micropinocytosis v. RME is valuable and well-done. So perhaps the latter.
--As suggested, the macropinocytosis figure has been moved from the supplement to the main paper – new Fig.6.

• As in the introduction, the wording of nucleation factor should be changed to nucleation promoting factors for clarity.
--Yes.

Reviewer #3: This is a well-executed study, showing the discovery and characterisation of a new WASP-family protein that is specific to a subset of mammals. The discovery of WHIMP is interesting to the field and has been well documented and presented here. The authors demonstrate that WHIMP is widely expressed in mouse tissues and cells and that it contributes to actin nucleation at the leading edge of cells. They link WHIMP also to dorsal ruffles, which contrubute to macropinocytosis and to src activation. This is a very intriguing new member of the WASP-family and the characterisation provided by this study is convincing and clear.
I have only minor comments.

1. Can the authors make it more clear in their abstract which organisms express WHIMP? e.g. is it a subset of mammals?
--We have clarified that WHIMP is encoded in a subset of mammals, and that we have only studied the mouse version in the current paper.

2. Is WHIMP near to WASP on the X chromosome? Did it likely arise from a gene duplication of WASP?
--*Whimp* is not near *Was* on the X. We now describe their respective positions (D vs A1.1) in Fig.S1. We guess that *Whimp* may have arisen from retrotransposition of another *Was*-family gene, but prefer not to speculate at this point.

3. Can the authors provide scans of original western blots (if they haven't already) as supplementary information.
--Yes, as requested, the paper's full blots were compiled into a 3-page Fig.S9.

4. Do the authors have the possibility to do quantitative RT-PCR to provide any insight into how abundant WHIPM is? The provide a yes/no gel image, which is OK, but if they can do Q-PCR it would be better.

--We agree that RT-PCR would be better, but the yes/no RT-PCRs were the only RNA work that we performed. Once we found that the WHIMP protein was expressed on immunoblots, all of our subsequent experiments involved working with the protein.
